# Effects of *Bauhinia forficata* Tea on Oxidative Stress and Liver Damage in Diabetic Mice

**DOI:** 10.1155/2016/8902954

**Published:** 2015-12-29

**Authors:** Andréia Caroline Fernandes Salgueiro, Vanderlei Folmer, Marianne Pires da Silva, Andreas Sebastian Loureiro Mendez, Ana Paula Pegoraro Zemolin, Thaís Posser, Jeferson Luis Franco, Robson Luiz Puntel, Gustavo Orione Puntel

**Affiliations:** ^1^Programa de Pós-Graduação em Bioquímica, Universidade Federal do Pampa, Campus Uruguaiana, BR 472 Km 585, Caixa Postal 118, 97508-000 Uruguaiana, RS, Brazil; ^2^Faculdade de Farmácia, Universidade Federal do Rio Grande do Sul, Avenida Ipiranga 2752, 90610-000 Porto Alegre, RS, Brazil; ^3^Programa de Pós-Graduação em Ciências Biológicas, Universidade Federal do Pampa, Campus São Gabriel, Avenida Antônio Trilha 1847, Centro, 97300-000 São Gabriel, RS, Brazil; ^4^Departamento de Morfologia, Universidade Federal de Santa Maria, Avenida Roraima 1000, Camobi, 97105-900 Santa Maria, RS, Brazil

## Abstract

This study was designed to evaluate the effects of *Bauhinia forficata* Link subsp. *pruinosa* (BF) tea on oxidative stress and liver damage in streptozotocin (STZ)-induced diabetic mice. Diabetic male mice have remained 30 days without any treatment. BF treatment started on day 31 and continued for 21 days as a drinking-water substitute. We evaluated (1) BF chemical composition; (2) glucose levels; (3) liver/body weight ratio and liver transaminases; (4) reactive oxygen species (ROS), lipid peroxidation, and protein carbonylation in liver; (5) superoxide dismutase (SOD) and catalase (CAT) activities in liver; (6) *δ*-aminolevulinate dehydratase (*δ*-ALA-D) and nonprotein thiols (NPSH) in liver; (7) Nrf2, NQO-1, and HSP70 levels in liver and pancreas. Phytochemical analyses identified four phenols compounds. Diabetic mice present high levels of NQO-1 in pancreas, increased levels of ROS and lipid peroxidation in liver, and decrease in CAT activity. BF treatment normalized all these parameters. BF did not normalize hyperglycemia, liver/body weight ratio, aspartate aminotransferase, protein carbonyl, NPSH levels, and *δ*-ALA-D activity. The raised oxidative stress seems to be a potential mechanism involved in liver damage in hyperglycemic conditions. Our results indicated that BF protective effect could be attributed to its antioxidant capacity, more than a hypoglycemic potential.

## 1. Introduction

Historically, basic therapy for treating several diseases includes the use of medicinal plants. Vegetable species with medicinal power have considered complex mixtures of biologically active products, and usually many of them are responsible for their biological properties [1]. Therefore, many plants considered medicinal have been used in folk medicine to treat* diabetes mellitus* (DM) [2]. Among these is* Bauhinia forficata* (BF) (Leguminosae, Fabaceae), popularly known as “paw of cow” [3].

In Brazil, the tea (infusion) of BF leaves is an important alternative treatment for people with DM [2]. The BF genus comprises about 300 species found especially in the tropical regions of the planet [3]. Besides their possible hypoglycemic potential, considerations about the antioxidant and hepatoprotective activities of some* Bauhinia* species have been postulated. For example, extracts of* Bauhinia forficata* Link and* Bauhinia cheilandra* showed antidiabetic activity in STZ and alloxan-induced diabetic rats [4–6]. Already, the antioxidant and hepatoprotective activity was previously demonstrated for* Bauhinia forficata* Link,* Bauhinia racemosa* Lam, and* Bauhinia variegata* [7–9]. However, we did not find in scientific literature studies with mice or rats that investigate the same* Bauhinia* species that we use here (*Bauhinia forficata* Link subsp.* pruinosa* (Vogel) Fortunato & Wunderlin).

Biological properties of* Bauhinia* species have been attributed to its phenolic compounds. In this context,* Bauhinia forficata* Link subsp.* pruinosa* are able to scavenge reactive oxygen species (ROS) because it contains flavonoids among its constituents (especially derivatives of quercetin and kaempferol) [10, 11]. These characteristics can be extremely important in diseases where there is an increase in oxidative stress, as in DM and its complications.

Indeed, chronic hyperglycemia in DM has related to a bigger ROS production and severe oxidative damage in different tissues, including the liver (for a review see [12]). Increased ROS has been known to induce changes in expression and activity of antioxidant enzymes superoxide dismutase (SOD) and catalase (CAT), as well as thiol oxidation and lipid peroxidation [12]. Furthermore, previous reports showed that, in experimental models of DM, the sulfhydryl-containing enzyme *δ*-aminolevulinate dehydratase (*δ*-ALA-D) was inhibited [13–15].

Moreover, increases in ROS production, both in liver and in pancreas, stimulate expression of factors related to cellular antioxidant response, such as NF-E2-related nuclear factor erythroid-2 (Nrf2), NADPH quinone oxidoreductase 1 (NQO-1), and heat shock protein 70 (HSP70) [16, 17]. According to Yeo et al. [18], antioxidant and chemical stress, including chemical DM induction in mice, increases NQO-1 expression.

Therefore, the aim of this study was to evaluate the effects of* Bauhinia forficata* Link subsp.* pruinosa* (Vogel) Fortunato & Wunderlin (infusion) (BF tea) treatment on oxidative stress and liver damage in diabetic mice. Among the parameters evaluated were the glycaemia, ROS production, lipid peroxidation, protein carbonylation, and nonprotein thiols levels in liver, as well as the activities of enzymes *δ*-ALA-D, SOD, and CAT in liver. Moreover, we evaluate the Nrf2, NQO-1, and HSP70 expression in liver and, additionally, in pancreas.

## 2. Materials and Methods

### 2.1. Chemicals

Sigma-Aldrich Chemical Co. (St. Louis, MO) supplied Ellman's reagent (DTNB) and streptozotocin. Labtest (Minas Gerais, Brazil) supplied commercial kits. Other reagents were obtained from local suppliers.

### 2.2. *Bauhinia forficata* (BF) Preparation

BF leaves were collected in September (spring) of 2014 in southern Brazil (29°44′58.8′′S 57°05′01.7′′W). Botanical identification of BF leaves samples was confirmed and a voucher specimen (number ICN 167491;* B. forficata* Link subsp.* pruinosa* (Vogel) Fortunato & Wunderlin) was deposited at ICN Herbarium of Federal University of Rio Grande do Sul (Brazil).

BF tea was prepared with naturally dried leaves in a 1 mg/mL proportion (weight of dried leaves/volume of water), described by Salgueiro et al. [11].

### 2.3. Chromatographic Analyses

Chromatographic analyses by HPLC were conducted, described in [11, 19], using a Prominence Liquid Chromatograph (Shimadzu, Kyoto, Japan). This apparatus is equipped with an SLC-10A controller, LC-20AD pump, SIL-10AF autosampler, and SPD-M10A PDA detector. An ODS-Hypersil Thermo Scientific C18 column (250 × 4.6 mm i.d., 5 *μ*m particle size) (Bellefonte, United States) was used. Mobile phase consisted of water containing 0.05% phosphoric acid (A) and acetonitrile (B) at a flow rate of 0.8 mL min^−1^ using the following gradients: 0.1–23 min, 10–40% of solvent B in A and 23.01–40 min, 10% solvent B, and 90% solvent A. Detection was done on a diode array detector (DAD) set at 340 nm and the injection volume was 20 *μ*L. The HPLC system was operated at 25 ± 1°C. Runs were made in triplicate. The reference standard chemical composition for BF tea was established previously by our group, identifying the compounds quercetin-3-O-(2-rhamnosyl) rutinoside, kaempferol-3-O-(2-rhamnosyl) rutinoside, quercetin-3-O-rutinoside, and kaempferol-3-O-rutinoside [11, 19].

### 2.4. *Diabetes Mellitus* (DM) Induction and BF Treatment

Committee on the Ethics of Animal Experiments approved this study (permit number 001/2012). All experiments were conducted with the minimum number of animals and in obedience to the guidelines for biomedical research stated by the Brazilian Societies of Experimental Biology. Animals were maintained in an enriched environment with a room-controlled temperature, 12 h light-dark cycle, and food and water available* ad libitum*.

Three-month male Swiss albino mice (30–35 grams) were divided into four different groups with six animals for each group:

1. Control received only citrate buffer intraperitoneally (*i.p.*) and drank water throughout the period.
2. DM received a single STZ dose (150 mg/kg)* i.p.* and drank water throughout the period.
3. BF received only citrate buffer* i.p.*, drank water during 30 days, and afterwards drank BF tea (1 mg/mL) during 21 days.
4. DM + BF received a single STZ dose (150 mg/kg)* i.p.*, drank water during 30 days, and afterwards drank BF tea (1 mg/mL) during 21 days.

STZ was freshly prepared in citrate buffer (0.05 M, pH 4.5), and before STZ administration the animals were fasted for a period of four hours. STZ dose was established, proposed by Animal Models of Diabetic Complications Consortium [20], in order to induce a severe hyperglycemia in mice. Five days after STZ injection the hyperglycemia was confirmed by collecting a tail drop of blood and using ACCU-Check Active (Roche Diagnostics) glucometer.

The BF concentration (approximately 313 mg/kg of body weight) was established after evaluation of liquid intake of diabetic mice in metabolic cage (9.4 ± 2.24 mL of tea per day) and body weight mean (0.030 kg). This dose is in accordance with previous studies that investigated the hypoglycemic and hepatoprotective activity of other* Bauhinia* species [4–6, 8, 9]. BF treatment started on day 31 and continued for 21 days in drinking water.

### 2.5. Tissue Preparation for Biochemical Analyses

After the period of treatment, animals were killed by cardiac puncture. This procedure was performed under enough ether anesthesia to ameliorate mice suffering. Mice livers were removed and carefully washed, and part of them were weighted and homogenized in 1 : 10 ratio of tissue to cold NaCl (0.9%). The homogenates were centrifuged at 4000 g for 10 min at 4°C and the supernatants (S1) collected for biochemical analyses. All the biochemical analyses were performed in the same day of euthanasia. The liver and body weight were used to evaluate the liver weight/body weight ratio.

### 2.6. Analysis of Glucose Levels and Liver Transaminases

Blood was collected in heparinized tubes by cardiac puncture after fasting for 6 hours. After centrifugation, levels of glucose and liver transaminases were determined in plasma using a commercial kit (Labtest, Minas Gerais/Brazil).

### 2.7. Assessment of 2,7-Dichlorofluorescein (DCFH) Oxidation

Indirect quantification of reactive oxygen species (ROS) production was determined in S1 samples by evaluation of dichlorofluorescein reactive species (DCF-RS) levels, proposed by Myhre et al. [21]. Briefly, an aliquot of S1 (100 *μ*L) were added to a medium containing Tris-HCl buffer (0.01 mM, pH 7.4) and DCFH-DA (7 *μ*M). This medium was incubated in the dark for 1 h until fluorimetric analysis (Ex: 488 nm; Em: 520 nm). The results were stated as DCF fluorescence intensity, corrected by protein content, and expressed as percentage of control.

### 2.8. Thiobarbituric Acid Reactive Species (TBA-RS) Levels

Lipid peroxidation was assayed by adding S1 samples (100 *μ*L) to a medium containing 8.1% sodium dodecyl sulfate, acetic acid buffer (pH 3.5), and 0.8% aqueous solution of thiobarbituric acid, proposed by Ohkawa et al. [22]. After heating at 95°C for 60 min, the red pigment produced was measured spectrophotometrically at 532 nm. The results were calculated using a standard curve constructed with malondialdehyde (MDA) at known concentrations and corrected by protein content. The results were expressed as nanomoles of MDA per milligram of protein.

### 2.9. Protein Carbonyl Levels

Protein carbonyl was measured in S1 samples, proposed by Levine et al. [23]. Briefly, an aliquot of S1 (200 *μ*L) were derivatized using 2,4-dinitrophenylhydrazine (DNPH). DNPH reaction proteins were precipitated with an equal volume of 20% (w/v) trichloroacetic acid and washed three times with an ethanol/ethyl acetate mixture (1 : 1). Finally, the precipitates were dissolved in 6 M guanidine HCl solution. Protein carbonyl levels were determined spectrophotometrically at 370 nm, against blanks. The results were calculated using the molar extinction coefficient of DNPH, corrected by protein content, and expressed as nanomoles of carbonyl per milligram of protein.

### 2.10. Superoxide Dismutase (SOD) Enzyme Activity

SOD enzyme activity was determined in S1 samples, proposed by Kostyuk and Potapovich [24]. This method is based on the capacity of SOD in inhibiting quercetin autooxidation. Briefly, S1 aliquots (25 *μ*L) were added to a medium containing 0.016 M phosphate buffer, 0.8 mM N,N,N',N'-Tetramethylethylenediamine, and 0.08 mM EDTA (final pH of the medium was 10). The kinetic analysis of SOD was measured spectrophotometrically at 406 nm after quercetin addition (1.5 mg of quercetin in 10 mL of N,N-Dimethylformamide). The results were corrected by protein content and expressed as unit per milligram of protein. One unit of SOD activity is defined as the amount of enzyme that inhibited the quercetin oxidation reaction by 50% of maximal inhibition. Fifty percent inhibition was produced by approximately 1.5 ng/mL of pure enzyme [17].

### 2.11. Catalase (CAT) Enzyme Activity

CAT enzyme activity was determined in S1 samples, proposed by Aebi [25]. This method is based on the rate of hydrogen peroxide (H_2_O_2_) degradation by the action of CAT. Briefly, S1 aliquot (20 *μ*L) was added to a medium containing potassium phosphate buffer (50 mM, pH 7.4) and H_2_O_2_ (1 mM). The kinetic analysis of CAT was measured spectrophotometrically at 240 nm after H_2_O_2_ addition. The results were calculated using the molar extinction coefficient of H_2_O_2_, corrected by protein content, and expressed as nmol H_2_O_2_/mg protein/min.

### 2.12. Delta-Aminolevulinic Acid Dehydratase (*δ*-ALA-D) Enzyme Activity

*δ*-ALA-D enzyme activity was determined in S1 samples, according to Sassa [26]. This method is based in analysis of porphobilinogen (PBG) formation after *δ*-aminolevulinic acid addition. Briefly, S1 samples (100 *μ*L) were mixed with *δ*-aminolevulinic acid (12 mM initial concentration). The tubes were incubated for 60 min at 37°C. The reaction was stopped by adding 10% trichloroacetic acid with 10 mM mercuric chloride. After centrifugation, an equal volume of Ehrlich reagent was added to the supernatant, and absorbance at 555 nm was recorded. The results were corrected by protein content and expressed as nanomoles of PBG per milligram of protein per hour of incubation.

### 2.13. Nonprotein Thiols (NPSH) Levels

NPSH levels were determined according to Ellman [27]. Briefly, the S1 samples were precipitated with 10% trichloroacetic acid (1 : 1) and centrifuged at 4000 g for 10 min at 4°C to obtain supernatants (S2). S2 (100 *μ*L) samples were added to a medium containing phosphate buffer (TFK 0.25 mM, pH 7.4), and Ellman reagent (DTNB 1 mM). The yellow pigment produced was measured spectrophotometrically at 420 nm. The results were calculated in relation to a standard curve constructed with glutathione (GSH) at known concentrations and corrected by protein content. The results were expressed as nanomoles of SH per milligram of protein.

### 2.14. Protein Content Determination

Protein content was determined in S1 samples, proposed by Bradford [28], and measured spectrophotometrically at 595 nm. Bovine serum albumin at known concentrations was used to construct a standard curve.

### 2.15. Western Blot Analysis

Western blotting was performed according to Posser et al. [29] with minor modifications. Part of the liver and pancreas were homogenized at 4°C in a medium containing 50 mM Tris, 1 mM EDTA, 0.1 mM phenylmethylsulfonyl fluoride, 20 mM Na_3_VO_4_, 100 mM sodium fluoride, and protease inhibitor cocktail (Sigma, MO), pH 7.0. The homogenates were centrifuged at 1000 g for 10 min at 4°C and the supernatants (S1) collected. After protein determination (following Bradford [28]) using bovine serum albumin as standard, *β*-mercaptoethanol and glycerol were added to samples to a final concentration of 8 and 25%, respectively, and the samples were frozen in −80°C until further analysis. Proteins (2 mg/mL) were separated using SDS-PAGE with 10% gels and then electrotransferred to nitrocellulose membranes as previously described [29]. Membranes were washed in Tris-buffered saline with Tween (TBST; 100 mM Tris-HCl, 0.9% NaCl, and 0.1% Tween-20, pH 7.5) and incubated overnight (4°C) with different primary antibodies (Santa Cruz Biotechnology, TX), all produced in rabbit (anti-Nrf2, anti-NQO-1, and anti-HSP70 anti-*β*-actin; 1 : 1000 dilution in TBST). Following incubation, membranes were washed in TBST and incubated for 1 h at 25°C with HRP-linked anti-rabbit-IgG secondary specific antibodies (Sigma, MO). The immunoblots were visualized in the Image Station 4000MM PRO using ECL reagent (Promega, WI). Immunoreactive bands were quantified using the Scion Image software and expressed as percentage of untreated controls.

### 2.16. Statistical Analysis

Data were expressed as mean ± SEM of the number of animals used in each experiment. Statistical analysis was performed using two-way ANOVA and Tukey post hoc test. Values of *p* < 0.05 were considered statistically significant. GraphPad prism 6 software was used for statistical analysis and for plotting graphs.

## 3. Results

### 3.1. Chromatographic Profile

**Figure 1:**
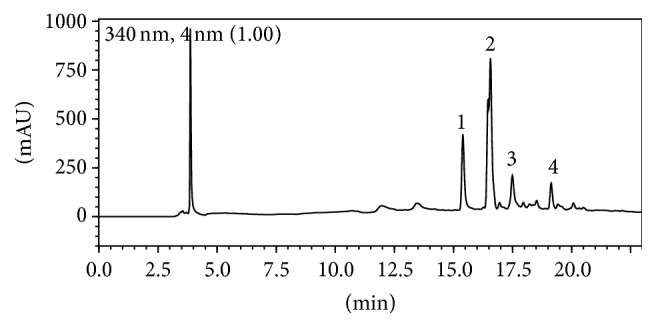
Chromatographic profile of* B. forficata* Link subsp.* pruinosa* (Vogel) Fortunato & Wunderlin tea. Chemical compounds identified Peak 1: quercetin-3-O-(2-rhamnosyl) rutinoside (retention time: 15.60 min); Peak 2: kaempferol-3-O-(2-rhamnosyl) rutinoside (retention time: 16.70 min); Peak 3: quercetin-3-O-rutinoside (retention time: 17.40 min); Peak 4: kaempferol-3-O-rutinoside (retention time: 19.10 min).

HPLC analysis of BF tea revealed the following main compounds kaempferol-3-O-(2-rhamnosyl) rutinoside (2) > quercetin-3-O-(2-rhamnosyl) rutinoside (1) > quercetin-3-O-rutinoside (3) > kaempferol-3-O-rutinoside (4) (Figure 1).

### 3.2. Glucose Levels

**Figure 2:**
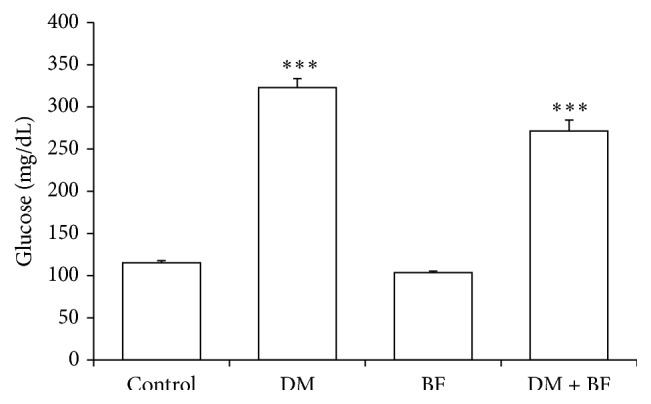
Glucose levels (mg/dL) at the end of treatment. The *∗* indicates significant difference in comparison to control group (*p* < 0.05).

Diabetic mice had a significant increase in the serum glucose levels, which were not reduced by BF (Figure 2).

### 3.3. Liver Toxicity Evaluation

**Figure 3:**
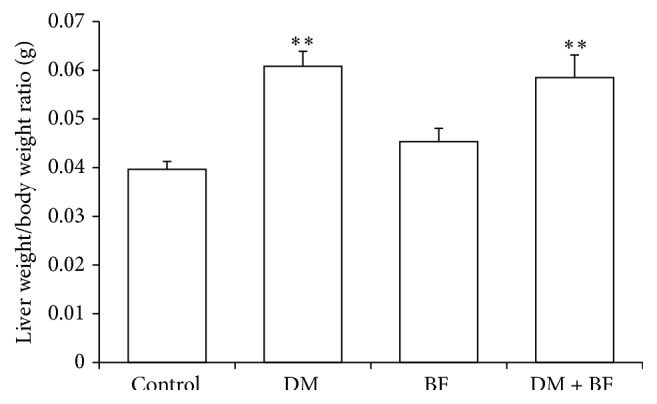
Liver/body weight ratio (g) of diabetic mice treated with BF. The *∗* indicates significant difference in comparison to control group (*p* < 0.05).

**Figure 4:**
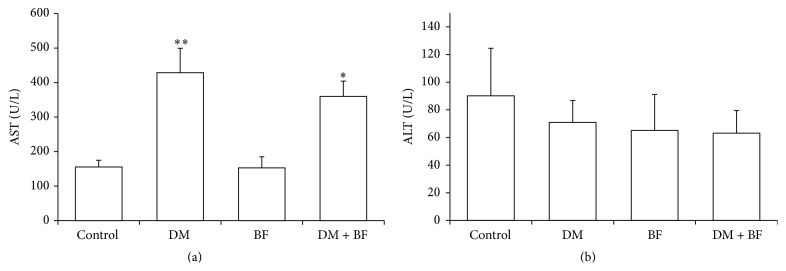
Alanine aminotransferase (a) and aspartate aminotransferase (b) levels (U/L) of diabetic mice treated with BF. The *∗* indicates significant difference in comparison to control group (*p* < 0.05).

The liver/body weight ratio was increased in diabetic mice when compared to control group. These changes were not modified by BF treatment. BF* per se* did not affect this parameter (Figure 3). Diabetic mice had a significant increase in aspartate aminotransferase (AST) level (Figure 4(a)) when compared to the control group. This change was not modified by BF treatment. Alanine aminotransferase (ALT) level (Figure 4(b)) was not changed by any treatment.

### 3.4. Liver Oxidative Stress Evaluation

**Figure 5:**
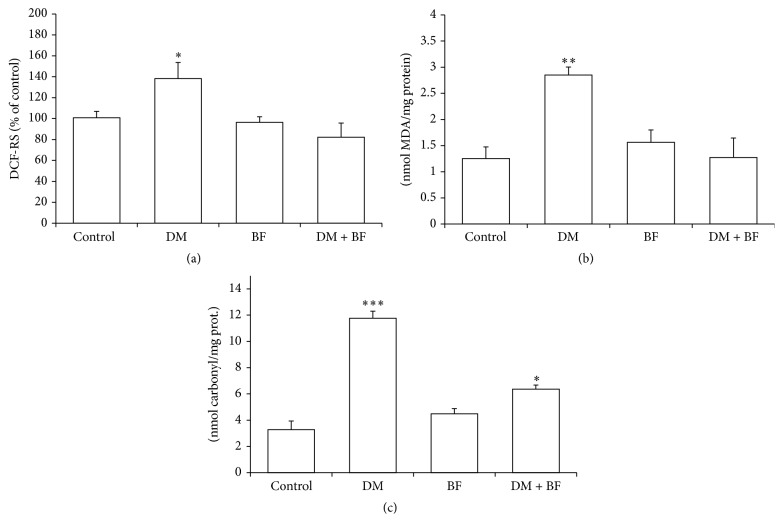
Liver dichlorofluorescein reactive species (DCF-RS) (a); thiobarbituric acid reactive species (TBA-RS) (b); and protein carbonyl levels (c) of diabetic mice treated with BF. The *∗* indicates significant difference in comparison to control group (*p* < 0.05).

BF treatment was effective in normalizing the increases in ROS (DCF-RS) and lipid peroxidation (TBA-RS) levels observed in diabetic mice, to the control levels (Figures 5(a) and 5(b), resp.). Furthermore, diabetic mice had an increase in the carbonylated protein levels (Figure 5(c)) that were only partially reduced by BF treatment.

**Figure 6:**
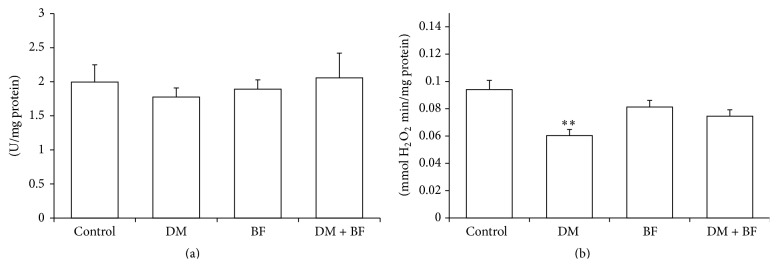
Liver superoxide dismutase (SOD) (a) and catalase (CAT) (b) activities of diabetic mice treated with BF. The *∗* indicates significant difference in comparison to control group (*p* < 0.05).

No difference in SOD activity was observed among the groups (Figure 6(a)). However, the diabetic mice had a significant decrease in CAT activity when compared to the control group. This decrease was attenuated by BF treatment (Figure 6(b)).

**Figure 7:**
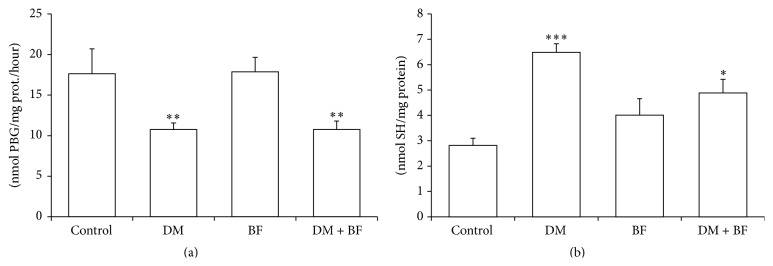
Liver delta-aminolevulinic acid dehydratase (*δ*-ALA-D) activity (a) and nonprotein SH (NPSH) levels (b) in diabetic mice treated with BF. The *∗* indicates significant difference in comparison to control group (*p* < 0.05).

The activity of liver *δ*-ALA-D was inhibited in diabetic mice. The inhibition of *δ*-ALA-D enzyme activity was not modified by BF treatment (Figure 7(a)). Addition of a thiol donor dithiothreitol (DTT) partially reactivated *δ*-ALA-D, however, without restoring the basal activity of *δ*-ALA-D (data not shown). The levels of nonprotein thiol groups (NPSH) were increased in diabetic mice and BF treatment restores only partially this increase (Figure 7(b)).

### 3.5. Western Blot Analysis

**Figure 8:**
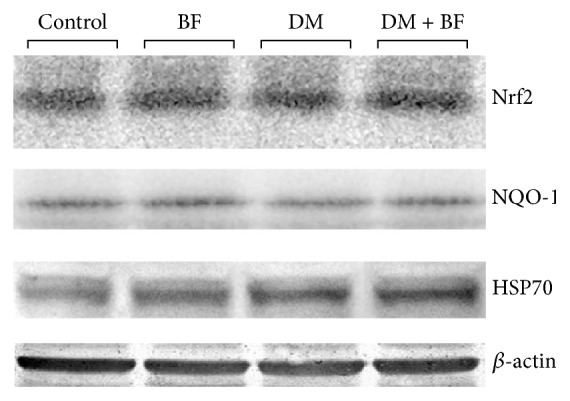
Liver Nrf2, NQO-1, and HSP70 protein levels in diabetic mice treated with BF. The data were normalized with *β*-actin expression and expressed as % of control.

**Figure 9:**
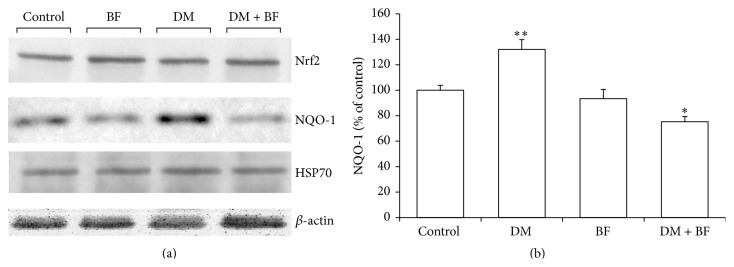
Pancreas Nrf2, NQO-1, and HSP70 protein levels in diabetic mice treated with BF (a). Graphical representation of NQO-1 pancreas protein levels (b). The data were normalized with *β*-actin expression and expressed as % of control. The *∗* indicates significant difference in comparison to the control group (*p* < 0.05).

Liver western blot showed that Nrf2, NQO-1, or HSP70 protein levels were not altered in diabetic mice or BF treatment when compared to the control (Figure 8). In pancreas, an increase in NQO-1 levels was observed, and BF treatment reduce these at levels lower than the control group (Figure 9). No differences in the levels of Nrf2 and HSP70 were observed among the groups in pancreas.

## 4. Discussion

The present study was designed to investigate the effects of* Bauhinia forficata* Link subsp.* pruinosa* (Vogel) Fortunato & Wunderlin (BF) tea against oxidative stress and liver damage in diabetic mice. In folk medicine, various species of BF have been used to treat* diabetes mellitus* (DM) [2], especially due to their possible hypoglycemic effect. Our results show BF tea reduced liver oxidative stress in diabetic mice, although it did not change the glycaemia.

In this context, the absence of hypoglycemic action of BF tea may be due to the nonextraction of some compounds in the aqueous fraction (infusion) or due to absence of kaempferitrin compound (kaempferol-3,7-O-(r)-dirhamnoside), pointed out as responsible for hypoglycemic action in other* Bauhinia* species [10].

Our results show BF tea* per se* does not determine abnormal hepatic growth or transaminases changes, indicating possible absence of toxicity (Figures 3 and 4(a), 4(b)). On the other hand, we had an increase in AST levels and in liver/body weight ratio in diabetic mice. The increase in liver/body weight ratio may be due to the reduction of body weight (data not shown), common in untreated diabetes [30]. Regarding transaminases, both AST and ALT are highly concentrated in the liver. However, ALT is localized only in the cellular cytoplasm, whereas AST is cytosolic in a minor portion and mitochondrial in a major portion. Furthermore, AST is highly concentrated in zone 3 of the hepatic acinus, and damage to this zone may indicate ischemic or toxic events, resulting in greater AST levels [31]. In case of diabetes, hepatic toxic events may occur in response to an excess in free fatty acids [32] results of insulin impairment. Known mechanisms for hepatic toxics events that increase transaminases levels in diabetic state include cell membrane disruption, mitochondrial dysfunction, toxin formation, oxidative stress, and recruited inflammatory cells [32].

We observe an increase in reactive oxygen species (ROS) and lipid peroxidation levels (Figures 5(a) and 5(b), resp.), indicating oxidative damage in liver. The assessment of DCF-RS is well accepted to determine ROS levels, as well as reactive nitrogen species able to oxidize the DCFH, a general index of oxidative stress. Similarly, TBA-RS assay is a known biomarker used to estimate lipid damage from cells and tissues, and its increased levels are an indirect evidence of high ROS production. Although BF tea treatment did not modify the changes in liver/body weight ratio and AST levels in diabetic mice, the plant was effective in reducing DCF-RS and TBA-RS levels. These findings reinforce our previously reported antioxidant activity of BF tea even at low concentrations [11]. The antioxidant activity of BF extracts has been attributed to high levels of polyphenols and flavonoids present in its composition [11, 33]. Here, we identify four major compounds (Figure 1) that were previously reported using liquid chromatography-electrospray ionization-mass spectrometry (LC-ESI-MS) [11, 19]. Among the chemical constituents identified in our extract, the quercetin and kaempferol derivate have been extensively studied to have antioxidant properties, such as reduction of TBA-RS levels and control of antioxidant response [10, 11].

Our results also showed that there is an increase in liver carbonylated protein levels (Figure 5(c)). This increase is reduced only partially by BF treatment, and it is not related with ROS levels that were controlled by BF treatment (Figure 5(a)). Probably, a longer BF treatment might reduce the protein carbonyl levels to control levels. This is possible whereas carbonylated proteins have a long half-life and take longer to suffer degradation when compared to normal proteins.

Concerning liver antioxidant enzymes, we observed a significant decrease in CAT activity in diabetic mice, which was reverted by BF tea treatment (Figure 6(b)). No changes were observed in liver SOD activity in diabetic mice. For instance, changes in antioxidant enzymes activities or its return to normal values following a previous decrease may occur as a compensatory mechanism in response to a constant exposure to increased oxidative stress, such as those determined by prolonged hyperglycemia. This could explain the decreases in SOD activity observed by some researchers and the normal SOD activity observed by other investigators (for a review see [12]).

We also observed a decrease in *δ*-ALA-D enzyme activity (Figure 7(a)), not related to a decrease in NPSH levels (Figure 7(b)), in diabetic mice. Several studies report that the *δ*-ALA-D enzyme activity is reduced in hyperglycemic conditions [13, 14, 34]. This occurs due to presence of thiol groups in its structure, which are sensitive to oxidation. This characteristic explains its use as a good oxidative stress biomarker [13, 14]. In diabetic mice, we observed an increase in thiol levels, probably due to a physiological compensatory effect. In this context, the NPSH levels, glutathione (GSH) as a major compound, increase to counteract the high ROS production [11]. GSH is a ubiquitous cellular three-peptide antioxidant that acts as an intracellular buffer being responsible for the maintenance of the thiol redox balance [35]. In this line, mainly three functional changes may lead to a *δ*-ALA-D enzyme activity reduction, namely, removal of divalent zinc from its catalytic site (1); oxidation of its critical thiol groups (2); or protein oxidation (3) [13, 14, 34]. Taking into account that there were no thiol levels compromising and that the SH donor dithiothreitol (DTT) only partially reactivated *δ*-ALA-D (data not shown) we believe that the mechanism of inhibition was linked to protein oxidation. In fact, reducing sugars can interact with critical lysine residues of *δ*-ALA-D catalytic site, oxidizing the lysine residues to disulfides and inactivating the enzyme [34]. In this context, inhibition of *δ*-ALA-D in diabetes may be related to hyperglycemia [13, 14].

Here, the oxidative damage in liver seems to occur without changes in Nrf2, HSP70, or NQO-1 protein levels (Figure 8). Different in pancreas (target organ of STZ), we observed changes in NQO-1 expression (Figure 9) that were minimized by BF treatment. The enzyme NQO-1 is generally considered as a detoxification enzyme and has been known to protect *β*-cells against stressors, including the diabetogenic agent STZ [18, 36]. There is evidence that NQO-1 knockout mice present increased pancreatic *β*-cell death induced by STZ [18]. Furthermore, both STZ and hyperglycemia have been known to increase ROS production [12], and NQO-1 enzyme plays an important role as a superoxide scavenger that may provide an additional level of protection against ROS toxicity [36]. The increase observed in pancreas NQO-1 could be associated with a possible response against the xenobiotic injury determined by STZ. However, more studies are necessary to highlight the reasons for increased expression of NQO-1 in pancreas but not in liver.

Although we did not observe changes in Nrf2 protein levels, the NQO-1 upregulation in pancreas and the elevated levels of GSH in liver suggest an early activation of Nrf2-antioxidant response element (ARE) pathway, probably in response to increase in ROS levels. In fact, under stressing condition, the transcription factor Nrf2 interacts with ARE and upregulates antioxidative genes including NQO-1, antioxidant enzymes, and GSH levels, which are very important components of the cellular antioxidant defense [37]. However, while NQO-1 is a stable protein (half-life greater than 18 hours in wild type cells) [38], Nrf2 is a highly unstable protein and its half-life is about 15 min under nonstress condition [39] to 100 min under stress condition [40]. In fact, according to Nguyen et al. [39], even in stress condition, Nrf2 has a short life and is still subject to a high rate of degradation. The same has been observed with the HSP70, which has a half-life of approximately 2 hours [41]. This rapid degradation rate occurs, presumably, to prevent its accumulation in an uncontrolled manner [39] and may be the reason why we cannot observe differences in the levels of this protein in our study.

We highlight that although our objective was to investigate effects of BF tea (crude aqueous extract) on liver damage in diabetic mice, some points are extremely relevant and deserve further attention in future investigations, in particular, deeper analysis of the pancreas, serum insulin concentration, analysis of BF compounds concentration in the plasma, and the role/effect of their isolated bioactive components.

## 5. Conclusion

Taken together, our observations suggested that diabetic mice present an increase in liver oxidative damage and in pancreas NQO-1 expression, which were modulated by BF treatment. Since BF tea decreased liver oxidative injury but does not change glycaemia, we believe that BF protective effect may be attributed especially to its antioxidant capacity.
